# Drug exposure misclassification in pharmacoepidemiology: Sources and relative impact

**DOI:** 10.1002/pds.5346

**Published:** 2021-09-07

**Authors:** Mirjam Hempenius, Rolf H. H. Groenwold, Anthonius de Boer, Olaf H. Klungel, Helga Gardarsdottir

**Affiliations:** ^1^ Division of Pharmacoepidemiology and Clinical Pharmacology, Utrecht Institute for Pharmaceutical Sciences Utrecht University Utrecht The Netherlands; ^2^ Department of Clinical Epidemiology Leiden University Medical Center Leiden The Netherlands; ^3^ Julius Center for Health Sciences and Primary Care University Medical Center Utrecht Utrecht The Netherlands; ^4^ Department of Clinical Pharmacy, Division Laboratory and Pharmacy University Medical Center Utrecht Utrecht The Netherlands; ^5^ Faculty of Pharmaceutical Sciences University of Iceland Reykjavik Iceland

**Keywords:** drug exposure, methodology, misclassification, pharmacoepidemiology

## Abstract

**Background:**

Drug exposure assessment based on dispensing data can be misclassified when patients do not adhere to their therapy or when information about over‐the‐counter drugs is not captured in the study database. Previous research has considered hypothetical sensitivity and specificity values, whereas this study aims to assess the impact of literature‐based real values of exposure misclassification.

**Methods:**

A synthetic cohort study was constructed based on the proportion of exposure theoretically captured in a database (range 0.5–1.0) and the level of adherence (0.5–1.0). Three scenarios were explored: nondifferential misclassification, differential misclassification (misclassifications dependent on an unmeasured risk factor doubling the outcome risk), and nondifferential misclassification in a comparative effectiveness study (RR_A_ and RR_B_ both 2.0 compared to nonuse, RR_A‐B_ 1.0).

**Results:**

For the scenarios with nondifferential misclassification, 25% nonadherence or 25% uncaptured exposure changed the RR from 2.0 to 1.75, and 1.95, respectively. Applying different proportions of nonadherence or uncaptured use (20% vs. 40%) for subgroups with and without the risk factor, an RR of 0.95 was observed in the absence of a true effect (i.e., true RR = 1). In the comparative effectiveness study, no effect on RR was seen for different proportions of uncaptured exposure; however, different levels of nonadherence for the drugs (20% vs. 40%) led to an underestimation of RR_A‐B_ (0.89).

**Discussion:**

All scenarios led to biased estimates, but the magnitude of the bias differed across scenarios. When testing the robustness of findings of pharmacoepidemiologic studies, we recommend using realistic values of nonadherence and uncaptured exposure based on real‐world data.

**Key Points**

- Drug exposure status retrieved from healthcare databases can be misclassified when patients do not adhere to therapy or when exposure information is not fully captured in the healthcare database.
- This simulation study assessed the impact of literature‐based realistic values of exposure misclassification on effect estimates for three scenarios: nondifferential misclassification, differential misclassification, and nondifferential misclassification in a comparative effectiveness study.
- In the scenarios studied, the proportions of nonadherence and uncaptured data or the differences in these values between subgroups needed to be relatively large to result in clinically relevant bias.
- We recommend the use of empirical‐based values of nonadherence and uncaptured exposure to test the robustness of findings of pharmacoepidemiologic studies.

## 1 INTRODUCTION

Observational studies on the safety and effectiveness of pharmacological agents are commonly performed using routinely collected data from administrative or healthcare databases. Examples include healthcare insurance databases, out‐patient pharmacy databases, and general practitioner (GP) databases. Information about drug exposure retrieved from these databases can usually only serve as a proxy for actual use (i.e., the patient ingesting the drug). Therefore, pharmacoepidemiologic research conducted using these databases is prone to exposure misclassification.

**FIGURE 1:**
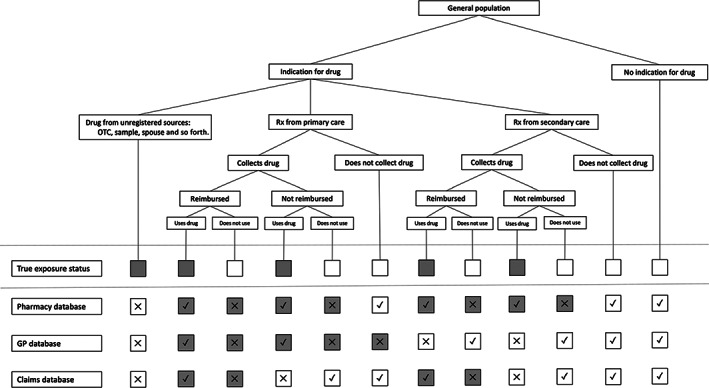
Sources of exposure misclassification in pharmacoepidemiology and the effects in different types of databases. Gray squares indicate “exposed”; white squares indicate “unexposed”. Rx, prescription; X, misclassified; √, correctly classified

The extent and nature of exposure misclassification differs per drug and per type of database that is used (see Table 1 and Figure 1). On the one hand, subjects may be misclassified as exposed to a specific drug based on a prescription or dispensing record in the database, when in fact they do not collect or administer the drug (nonadherence).^1^ For example, nonadherence to antidepressants is estimated between 10% and 35%.^2^, ^3^, ^4^, ^5^, ^6^, ^7^ On the other hand, subjects can be misclassified as nonexposed when information about the exposure is not captured in the database.^1^, ^8^, ^9^ This type of misclassification can occur for over‐the‐counter (OTC) drugs, drug samples, drugs with a restrictive drug coverage policy, use of drugs that were originally prescribed to someone else, or use of drugs that are prescribed in a clinical setting that is not captured in the database being used. The sources and extent of uncaptured exposure depend on the drug being studied and the database that is being used for a study, as described in Table 1 and Figure 1.

**TABLE 1:** Sources of exposure misclassification in the different databases

	Single prescriber database	Pharmacy dispensing database	Claims database
Exposed misclassified as unexposed	Drug prescribed by another prescriber	Drug bought as OTC	Drug bought as OTC
	Drug bought as OTC, without prescription	Drug sample	Drug sample
	Drug sample		Use of drugs that were originally prescribed to someone else
	Use of drugs that were originally prescribed to someone else	Use of drugs that were originally prescribed to someone else	Drug not reimbursed
Unexposed misclassified as exposed	Drug not collected at pharmacy	Drug collected, but not ingested	Drug collected, but not ingested
	Drug collected, but not ingested		

Reporting guidelines for pharmacoepidemiologic studies indicate that exposure misclassification should always be discussed.^10^, ^11^ Although this is generally the case, the quantification of the potential impact of exposure misclassification is uncommon.^12^ This is problematic, since nondifferential misclassification of binary exposure variables leads to bias toward the null and may lead to associations not being detected, especially if the effect under study is small. In addition, misclassification can be associated with patient characteristics, such as age,^2^, ^4^, ^5^, ^13^, ^14^, ^15^, ^16^, ^17^, ^18^ sex,^4^, ^13^, ^19^ socio‐economic status,^3^, ^5^, ^13^, ^15^, ^16^ and medical burden^16^, ^19^—characteristics that are often also related to the risk of the outcome. Since this could lead to differential exposure misclassification, thus causing bias toward or away from the null, the potential impact of such misclassification is not trivial.

**FIGURE 2:**
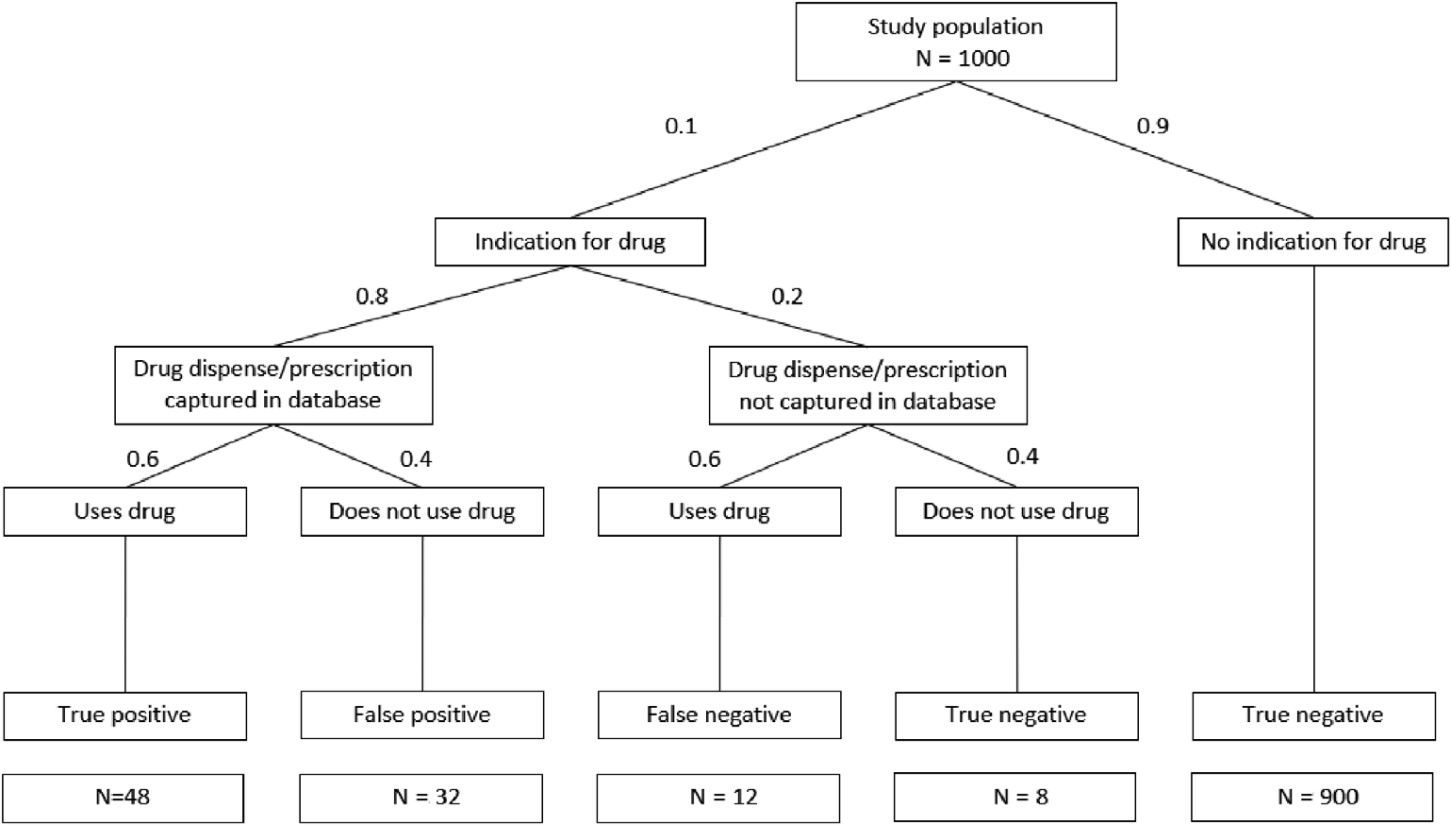
Model used for simulation analysis, with values for exposure prevalence (10%), nonadherence (40%) and uncaptured data (20%) and the corresponding exposure status. Sensitivity is in this example: True Positive/(True Positive + False Negative) = 48/(48 + 12) = 0.80; Specificity is True Negative/(True Negative + False Positive) = 908/(908 + 32) = 0.966

Key measures to quantify misclassification are sensitivity and specificity.^20^ Sensitivity is calculated as the proportion of exposed subjects who are classified as being exposed: True positive/(True positive + False Negative). Specificity is defined as the proportion of unexposed subjects who are classified as being unexposed: True Negative/(True Negative + False Positive). The effect of uncaptured exposure and nonadherence on sensitivity and specificity is illustrated with a numerical example in Figure 2.

Sensitivity is directly related to the proportion of exposure that is captured; an 80% captured exposure equals a sensitivity of 0.8. The value of specificity is affected by both nonadherence and exposure prevalence. A lower exposure prevalence will result in a higher proportion of truly unexposed subjects and thus a higher specificity. For example, 20% nonadherence to a drug with 10% prevalence results in a specificity of 0.987, while the specificity decreases to 0.867 when the exposure prevalence is 50%. On the other hand, in a situation of 10% exposure prevalence, 40% nonadherence results in a specificity of 0.966, compared to 0.987 for 20% nonadherence—both specificity values are high but relate to large differences in adherence rates (Figure 2).

It is therefore important to substantiate the values for sensitivity and specificity with known values of exposure prevalence, nonadherence, and uncaptured exposure to apply realistic scenarios in assessing the impact of exposure misclassification. Small deviations in specificity can imply large differences in adherence when the exposure prevalence is low.

In pharmacoepidemiology, to date, research into the impact of exposure misclassification on effect estimates has focused on individual sources of misclassification, such as nonadherence or reimbursement status,^21^ or applied hypothetical values to sensitivity and specificity that are not always supported by real data regarding adherence and the proportion of exposure that is captured in the study database.^20^, ^22^, ^23^, ^24^, ^25^ This study therefore aims to assess the impact of literature‐based realistic values of nonadherence and uncaptured use in simulated data, to investigate the relative impact of both sources of exposure misclassification.

## 2 METHODS

We constructed synthetic datasets of patient cohorts based on predefined exposure prevalence, the proportion of exposure that is captured in the database, and the level of adherence. Patients were divided into four different groups based on their exposure classification: true positive (observed definition as “exposed” is correct), true negative (observed definition as “nonexposed” is correct), false positive (observed definition as “exposed” is incorrect due to nonadherence), and false negative (observed definition as “nonexposed” is incorrect when information about exposure is not captured in database). Outcomes were subsequently assigned as a function of the baseline risk and the relative risk of exposure based on the actual exposure status. Observed relative risks were calculated based on the observed exposure status.

We explored the impact of nonadherence and uncaptured data in three scenarios: nondifferential exposure misclassification, differential exposure misclassification, and nondifferential exposure misclassification when comparing two drugs. We then applied this to two real‐world examples to further understand the impact of the different sources of exposure misclassification. Details of these scenarios are described below.

## 3 CONCEPTUAL SCENARIOS

### 3.1 Nondifferential exposure misclassification

In the first scenario, we investigated the extent to which nondifferential exposure misclassification could cause bias toward the null. In this scenario, exposure to Drug A was compared with nonexposure. Different levels of nonadherence (0.10, 0.25, and 0.50) and uncaptured exposure (0.10, 0.25, and 0.50) were applied, both separately and in combination. These values were chosen based on the range of values for nonadherence and uncaptured data found in the literature (Table 2). Different levels of true exposure prevalence were used (pr_true_ 0.01, 0.10, and 0.25), again based on the values described in the literature. The observed exposure prevalence (pr_obs_) was calculated to achieve this true exposure prevalence, accounting for the level of adherence (pr_obs_ = pr_true_/adherence). A baseline risk of 0.1 of the outcome and relative risks of 1.25, 2.0, and 5.0 of the exposure effect were investigated, and observed relative risks were calculated. The percentage bias was calculated as follows: %bias = [log(RR_obs_) – log(RR_true_)]/log(RR_true_) × 100%. In addition, the sensitivity and specificity of the exposure assessment were also calculated based on both the true and the observed exposure statuses.

**TABLE 2:** Basic parameters for the two scenarios

	NSAIDs values (literature reference)		Antidepressant agent values (literature reference)	
Proportion of general population receiving prescription	0.25 (0.04–0.58)^26^, ^27^, ^28^, ^29^, ^30^, ^31^, ^32^		0.10 (0.05–0.20)^29^, ^33^	
Of which from GP	0.85^34^		0.85 (0.75–0.90)^18^, ^35^, ^36^, ^37^, ^38^	
Proportion filling prescription	0.95 (0.91–0.95)^7^, ^39^, ^40^		0.80 (0.65–0.95)^2^, ^3^, ^4^, ^5^, ^6^, ^7^	
Proportion actual starts using drug^a^	0.95^40^		0.80 (0.60–0.80)^6^, ^16^, ^18^, ^35^, ^41^	
Proportion users that buy drug OTC	0.5 (0.5–0.9)^26^, ^27^, ^28^, ^42^		NA	
Baseline risk on (gastrointestinal) bleeding	0.01^43^ (10‐year risk)		0.025^44^	
Observed relative risk	3.5 (2.5–4.5)^43^		1.4^44^	
Reimbursement	Only on prescription^45^, ^46^		Full	
Differential misclassification (old vs. young)	Old: Baseline risk: 0.02^43^ Captured: 0.75^42^	Young: Baseline risk: 0.01^43^ Captured: 0.25^42^	Old: Baseline risk: 0.05 Adherence: 0.80^4^, ^5^	Young: Baseline risk: 0.025^44^ Adherence 0.70^4^, ^5^
Comparative effectiveness (drug A vs. drug B)	Meloxicam: Relative risk: 4.0^47^ Captured: 0.85^34^	Diclofenac: Relative risk: 4.0^47^ Captured: 0.50^26^, ^27^, ^28^, ^42^	Escitalopram: Relative risk: 1.5^44^ Adherence: 0.80^19^	Paroxetine: Relative risk: 1.5^44^ Adherence: 0.60^19^

^a^ The percentages found in these studies are predominantly defined as having only one prescription dispensed. The numbers from these studies comprise thus both patients that do not initiate the use and these that discontinue the use early.

### 3.2 Differential exposure misclassification

In the second scenario, we investigated the extent to which differential exposure misclassification could cause bias away from the null. For this scenario, it was assumed that the exposure did not influence the risk of the outcome (RR_true_ 1.0), but that the presence of a binary risk factor had an impact on both the amount of exposure misclassification (i.e., the level of nonadherence and uncaptured data) and the risk of the outcome (RR 1.5 and 2.0). This binary risk factor was present in 50% of all subjects.

Exposure to Drug A was compared with nonexposure, the exposure prevalence (pr_true_) was 0.1, and the baseline risk of the outcome was 0.1. Differences in the level of nonadherence and the proportion of uncaptured prescriptions between subjects with and without the risk factor that would result in an observed relative risk of 0.80, 0.90, 1.10, or 1.25 were plotted.

### 3.3 Comparative effectiveness research (CER): Drug A versus Drug B

In the third scenario, we examined the extent to which differences in the degree of nondifferential exposure misclassification between two study drugs could cause bias away from the null. In this scenario, exposure to Drug A was compared with exposure to Drug B. Both drugs were considered to increase the risk of the outcome compared to nonuse (either both RR 1.5 and both RR 2.0, with a baseline risk of 0.1), resulting in an RR_A‐B_ of 1.0. The exposure misclassification was considered nondifferential, but different levels of adherence and the proportion of prescriptions that were captured were applied for Drugs A and B. Nonadherence to Drugs A or B would place individuals in the nonuser category, not in the other category of exposure. The exposure prevalence (pr_true_) was 0.1 for both drugs.

Differences in the levels of nonadherence between Drug A and Drug B that would result in an observed relative risk of 0.80, 0.90, 1.10, or 1.25 were plotted. This was also done for differences in the proportion of uncaptured prescriptions.

## 4 APPLICATION IN TWO CASE STUDIES

In addition to the conceptual scenarios, two real‐life examples were investigated (Table 2).

### 4.1 Nonsteroidal anti‐inflammatory drugs (NSAIDs) and the risk of gastrointestinal bleeding

The first example focused on the relation between exposure to NSAIDs and the risk of gastrointestinal bleeding. The baseline risk of gastrointestinal bleeding is 0.01 per 10 person‐years.^43^ NSAIDs can, however, damage the protective gastric mucus layer via different mechanisms, thereby increasing the risk of gastrointestinal bleeding,^48^ which occurs most often immediately after administration.^49^ Adherence to NSAIDs is usually quite high (~95%), since patients take it for symptom relief.^7^, ^39^, ^40^

In most countries, some NSAIDs are only accessible through a prescription, while other NSAIDs are available OTC. In the Netherlands, for example, meloxicam is only available through a prescription, whereas diclofenac is available OTC. In the case of OTC NSAIDs, approximately 50% of their use is without a prescription.^26^, ^27^, ^28^, ^42^ OTC use of NSAIDs varies for different age categories: 75% of younger subjects (18–20 years) obtain their NSAIDs without a prescription (i.e., OTC), compared to 25% in those aged 65 years.^42^ In addition, the risk of a gastrointestinal ulcer increases with age and is twice as high for subjects aged 75 years or older, compared to younger subjects.^50^ The relative risk of gastrointestinal bleeding from meloxicam and diclofenac is comparable (RR ~4.0).^47^

### 4.2 Selective serotonin reuptake inhibitors (SSRIs) and the risk of bleeding

The second example concerned the relation between exposure to SSRIs and the risk of severe bleeding. The baseline risk of severe bleeding is about 0.025.^44^ SSRIs inhibit the platelet serotonin transporter, causing platelets to release less serotonin and hindering the vasoconstriction and aggregation of platelets,^51^ resulting in approximately a 1.5 times higher risk of bleeding.^44^ SSRIs and other antidepressant drugs are prescription‐only drugs, predominantly prescribed by GPs, although they can be prescribed by specialists as well.^18^, ^35^, ^36^, ^37^, ^38^ Nonadherence is known to be quite high for antidepressant drugs, with ~20% not filling in the first prescription.^2^, ^3^, ^4^, ^5^, ^6^, ^7^ In addition, even when patients do fill their prescription, a large proportion of them do not initiate treatment.^6^, ^16^, ^18^, ^35^, ^41^ The level of nonadherence can differ between the individual SSRIs. For this case study, we assumed nonadherence to be twice as high for paroxetine as compared to escitalopram.^19^ The level of nonadherence also differs between different age categories and is roughly 1.5 times higher in younger subjects (<=65 years) than those >65 years.^4^, ^5^ As mentioned before, the risk of bleeding is increased in older subjects (RR 2.0).^50^

For both examples, we calculated the underlying relative risk that would generate the observed relative risk in case of nondifferential misclassification, given the known numbers for uncaptured exposure and nonadherence (Table 2). Then, we compared meloxicam and diclofenac with a different proportion of captured exposure, and we compared escitalopram with paroxetine with different levels of adherence. Finally, we divided the cohort into two groups, namely, “old” and “young,” with different levels of uncaptured exposure and nonadherence and different risks of the outcome, and we calculated crude relative risks with and without correcting for the age effect.

## 5 RESULTS

### 5.1 Nondifferential exposure misclassification

**FIGURE 3:**
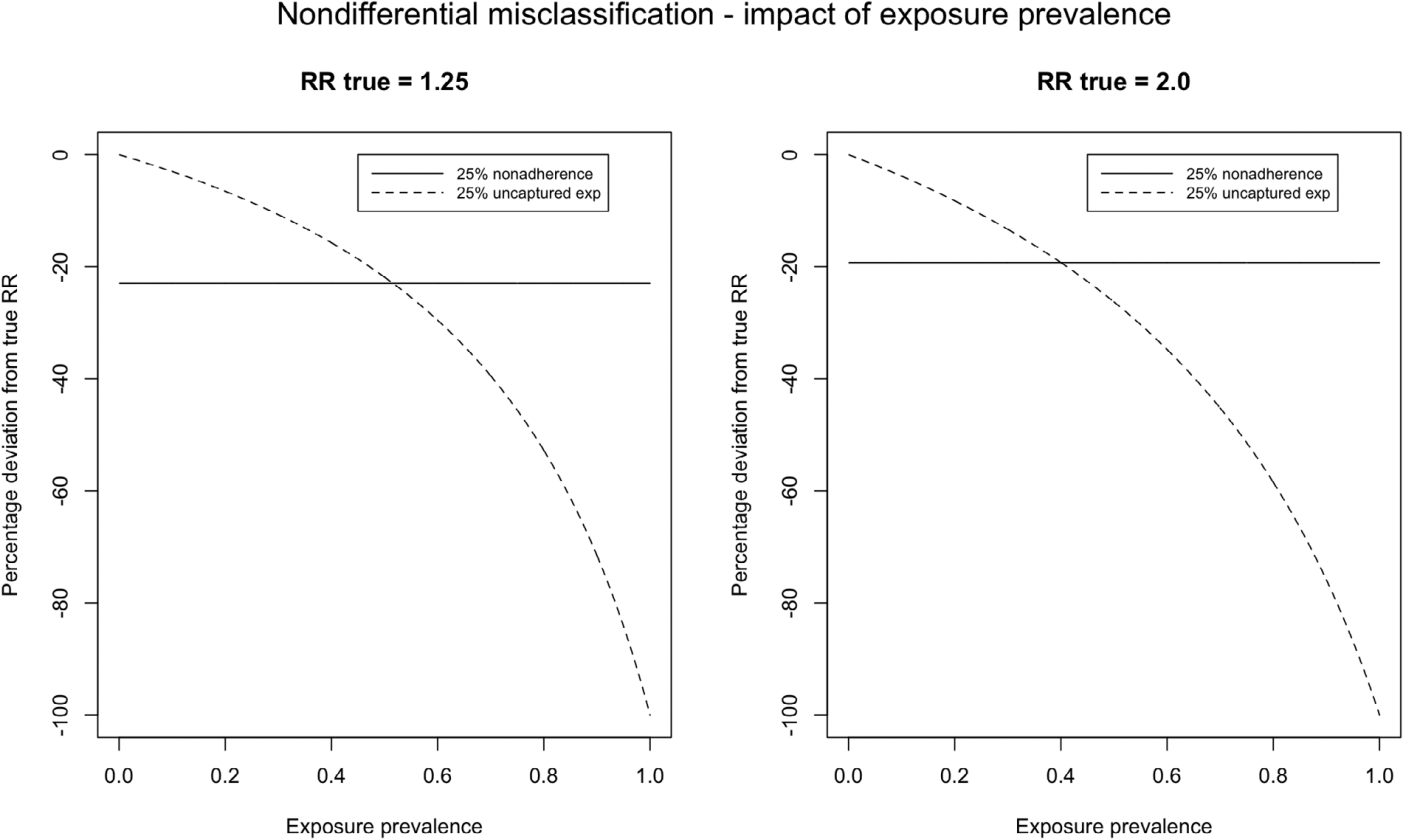
Percentage deviation from the true RR with 25% nonadherence or 25% not‐captured data against different exposure prevalences

The results of the analysis with nondifferential exposure misclassification are presented in Table 3. Nonadherence generally had a greater impact on RR than uncaptured exposure. For example, for a drug with a prevalence of 0.1, applying 25% nondifferential nonadherence to our model changed the RR from 2.0 to 1.75 (% deviation −19.3% on log[RR] scale) while applying 25% nondifferential uncaptured exposure changed the RR from 2.0 to 1.95 (−3.9%). With increasing prevalence of exposure, the effect of uncaptured exposure did, however, increase, while the effect of nonadherence did not. For exposure with a prevalence greater than 40%–50%, the effect of uncaptured data was greater than the effect of nonadherence (Figure 3). The largest effect was observed for the scenario with an exposure prevalence of 25%, 50% nonadherence, 50% uncaptured exposure, and a relative risk of 5.0. In this scenario, the relative risk changed to 1.8—a decrease of 65.5%. In Appendix S1, more extensive tables are presented (Tables S1a‐d), detailing the impact of different values for nonadherence and uncaptured data for different exposure prevalences.

**TABLE 3:** The effect of non‐differential exposure misclassification due to data that is uncaptured or nonadherence on the effect estimates

						RR 1.25		RR 2.0		RR 5.0	
True exp prevalence	Uncaptured	Nonadherence	Sens	Spec		RR obs	% dev	RR obs	% dev	RR obs	% dev
0.01	0.1	1	0.9	1.00		1.25	−0.13	2.00	−0.20	4.98	−0.50
0.1	0.1	1	0.9	1.00		1.25	−1.37	1.98	−2.20	4.79	−5.28
0.25	0.1	1	0.9	1.00		1.24	−4.00	1.94	−6.25	4.43	−14.29
											
0.01	0.25	1	0.75	1.00		1.25	−0.40	2.00	−0.50	4.95	−1.25
0.1	0.25	1	0.75	1.00		1.24	−3.36	1.95	−5.30	4.51	−12.20
0.25	0.25	1	0.75	1.00		1.23	−9.43	1.86	−14.29	3.82	−29.41
											
0.01	0.5	1	0.5	1.00		1.25	−0.80	1.99	−1.00	4.90	−2.50
0.1	0.5	1	0.5	1.00		1.23	−6.40	1.90	−10.00	4.13	−21.75
0.25	0.5	1	0.5	1.00		1.21	−17.24	1.75	−25.00	3.18	−45.45
											
0.01	1	0.1	1	1.00		1.23	−10.00	1.90	−10.00	4.60	−10.00
0.1	1	0.1	1	0.99		1.23	−10.00	1.90	−10.00	4.60	−10.00
0.25	1	0.1	1	0.97		1.23	−10.00	1.90	−10.00	4.60	−10.00
											
0.01	1	0.25	1	1.00		1.19	−25.00	1.75	−25.00	4.00	−25.00
0.1	1	0.25	1	0.97		1.19	−25.00	1.75	−25.00	4.00	−25.00
0.25	1	0.25	1	0.92		1.19	−25.00	1.75	−25.00	4.00	−25.00
											
0.01	1	0.5	1	0.99		1.13	−50.00	1.50	−50.00	3.00	−50.00
0.1	1	0.5	1	0.89		1.13	−50.00	1.50	−50.00	3.00	−50.00
0.25	1	0.5	1	0.67		1.13	−50.00	1.50	−50.00	3.00	−50.00
											
0.01	0.1	0.1	0.1	1.00		1.23	−10.00	1.90	−10.19	4.58	−10.46
0.1	0.1	0.1	0.1	0.99		1.22	−11.20	1.88	−12.09	4.40	−14.89
0.25	0.1	0.1	0.1	0.97		1.21	−14.05	1.84	−16.13	4.06	−23.53
											
0.01	0.25	0.25	0.25	1.00		1.19	−25.20	1.75	−25.44	3.96	−26.00
0.1	0.25	0.25	0.25	0.97		1.18	−28.28	1.70	−29.73	3.60	−35.00
0.25	0.25	0.25	0.25	0.92		1.16	−34.69	1.62	−38.46	3.00	−50.00
											
0.01	0.5	0.5	0.5	0.99		1.12	−50.57	1.49	−50.75	2.94	−51.49
0.1	0.5	0.5	0.5	0.94		1.11	−56.16	1.42	−57.89	2.45	−63.64
0.25	0.5	0.5	0.5	0.83		1.08	−68.00	1.29	−71.40	1.80	−80.00

Applying the scenario of nondifferential exposure misclassification to the example of NSAIDs and the risk of bleeding, assuming 50% uncaptured exposure and 5% nonadherence, we found that when an RR of 3.5 was observed, the true RR was 6.3 (−32.0%). For the case study of SSRIs, we found that when an RR of 1.40 was observed, the true RR was 1.51 (−18.4%), assuming 20% uncaptured exposure, and 20% nonadherence.

### 5.2 Differential exposure misclassification

**FIGURE 4:**
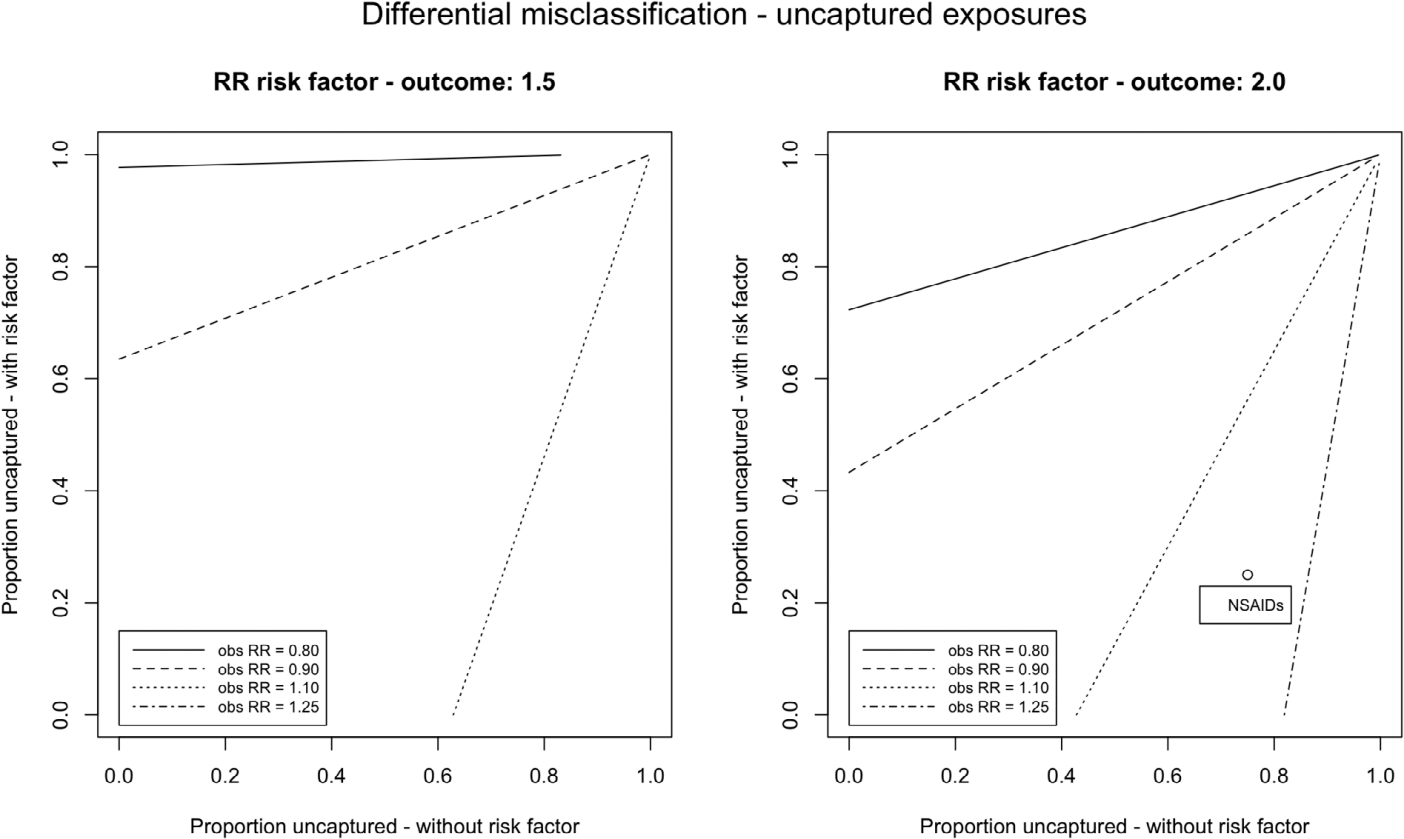
Observed relative risks obtained with different proportion of exposure captured for subjects with and without risk factor, in absence of a true effect (RR_true_ = 1.0). Observed relative risk for differential misclassification to NSAIDs caused by age was 1.18, assuming a relation of RR 2.0 for older subjects compared to younger subjects, 25% captured exposures for younger subjects, and 75% captured exposures for older subjects

Figure 4 illustrates the different proportions of uncaptured exposure for subjects with or without the risk factor required to observe an RR of 0.80, 0.90, 1.10, or 1.25 in the absence of a true relationship between exposure and outcome (RR_true_ = 1.0). For example, if a risk factor increases the risk of the outcome by a factor of 2, and if 90% and 50% of the exposure for subjects with and without this risk factor, respectively, were recorded in the database, then the resulting observed RR was 1.10. Moreover, with 80% and 60% captured exposure, respectively, an RR of 1.05 would have been observed. If the risk factor instead increased the risk of the outcome by a factor of 1.5, then an RR of 1.03 would have been observed.

**FIGURE 5:**
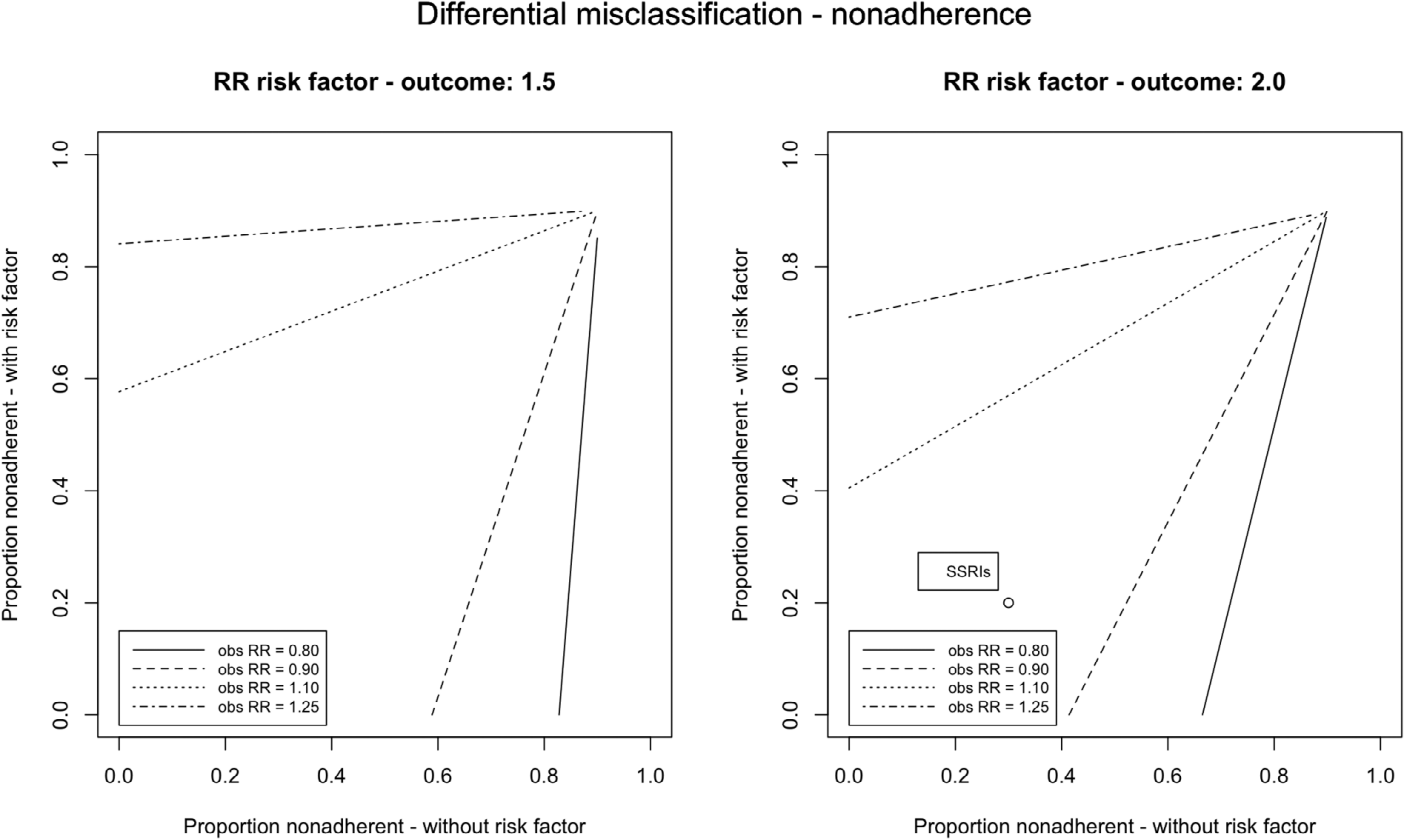
Observed relative risks obtained with different levels of nonadherence for subjects with and without confounding risk factor, in absence of a true effect (RR_true_ = 1.0). Observed relative risk for differential misclassification to SSRIs caused by age was 1.05, assuming a relation of RR 2.0 for older subjects compared to younger subjects, 30% nonadherence for younger subjects, and 20% nonadherence for older subjects

The results for the different levels of adherence are depicted in Figure 5. Approximately the same patterns were found for different levels of adherence for subjects with and without the risk factor: with 50% adherence for subjects with the risk factor and 90% adherence for subjects without this risk factor, the resulting observed RR was found to be 1.12. If the risk factor had a stronger effect on the outcome, then the effects were more pronounced. Stratification on the risk factor removed the effect of the differential misclassification in both situations. In Appendix S1, more figures are presented, illustrating the impact of differential exposure misclassification with different proportions of subjects with the risk factor and different relative risks between the risk factor and the outcome.

In case of a relation between NSAIDs and gastrointestinal bleeding, the risk factor “age” was considered to increase the risk of the outcome by a factor of 2, and the proportion of captured exposure was 75% and 25% for the “old” and “young” subjects. In this case, a relative risk of 1.18 could have been observed instead of 1.0, when no correction for this risk factor would have been applied (Figure 4). Stratification on age resulted in a relative risk of 1.0 in both subgroups.

For the case study of SSRIs, different levels of adherence were applied for the “old” and “young” subjects (80% vs. 70% respectively). When the risk factor “age” was again considered to increase the risk of the outcome by a factor of 2, there was only a small deviation from the true effect (RR 0.97 instead of 1.0; Figure 5). Again, stratification on age resulted in a relative risk of 1.0 in both subgroups.

### 5.3 CER

When two drugs were compared with each other, no effect of different levels of captured exposure was seen, as this resulted in sampling of all exposed subjects. As long as this occurred randomly, the risks remained the same, as did the risk ratio. This is illustrated in Table 4, with the case study of meloxicam and diclofenac.

**TABLE 4:** Impact of different values of uncaptured data in comparative effectiveness research

	Truth		Observed data	
Diclofenac use versus nonusers	Drug D1	Drug D0	Drug D1	Drug D0
Y = 1	6000	8500	3000	11 500
Y = 0	9000	76 500	4500	81 000
Total	15 000	85 000	7500	92 500
Risk	0.4	0.1	0.4	0.124
RR	4.0		3.2	
Meloxicam versus nonusers	Drug M1	Drug M0	Drug M1	Drug M0
Y = 1	200	9950	200	9950
Y = 0	300	89 550	300	89 550
Total	500	99 500	500	99 500
Risk	0.4	0.1	0.4	0.1
RR	4.0		4.0	
Diclofenac versus meloxicam	Drug D1	DrugM1	Drug D1	DrugM1
Y = 1	6000	200	3000	200
Y = 0	9000	300	4500	300
Total	15 000	500	7500	500
Risk	0.4	0.4	0.4	0.4
RR_D−M_	1.0		1.0	

*Note*: Values used: baseline risk: 0.1; RR: diclofenac 4.0, meloxicam 4.0; exposure prevalence: diclofenac 0.10, meloxicam 0.005; data capture: diclofenac 0.5, meloxicam 1.0; adherence: diclofenac 1.0, meloxicam 1.0.

Abbreviations: D1, exposed to diclofenac; M1, exposed to meloxicam; P0 and E0, nonexposed to diclofenac or meloxicam; Y, outcome.

**FIGURE 6:**
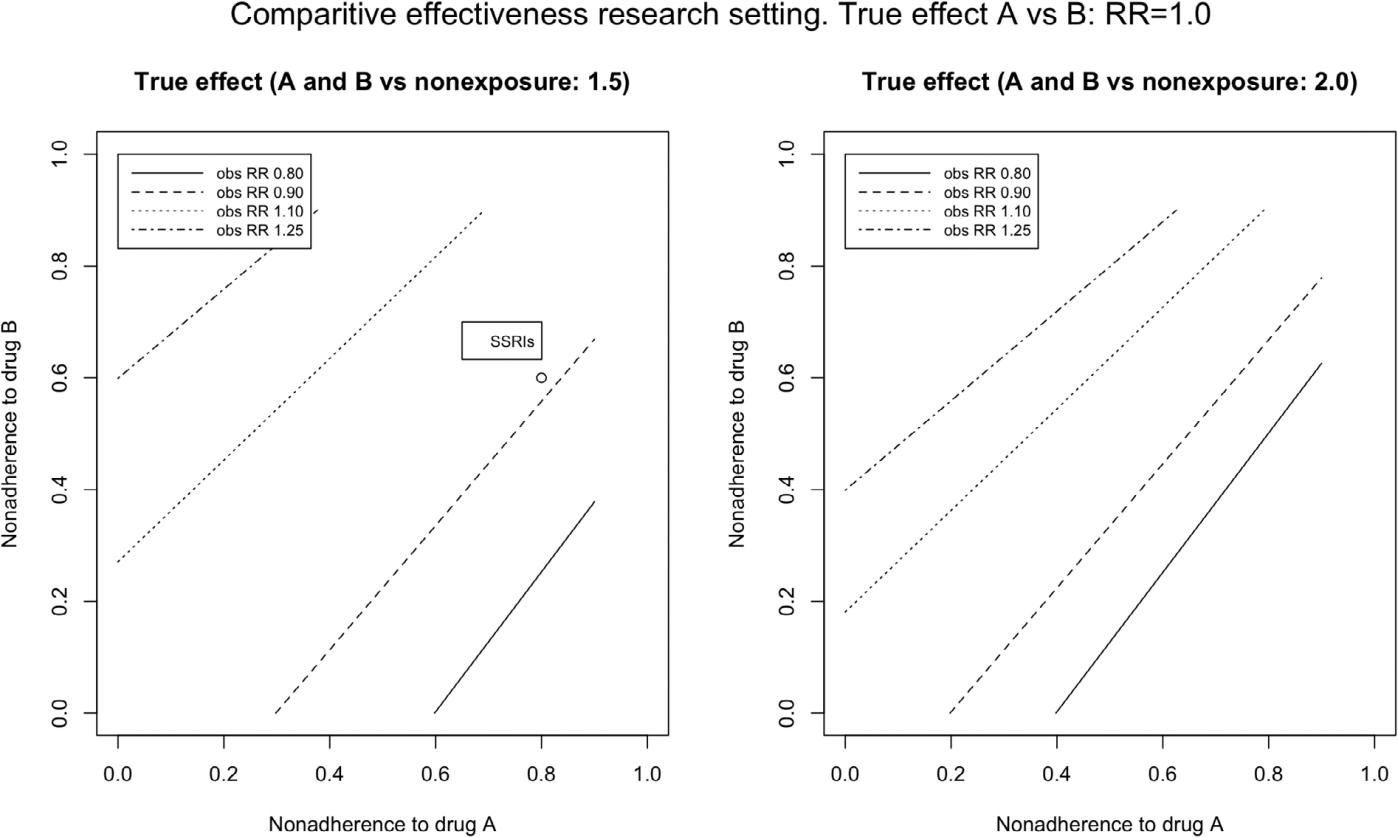
Observed relative risks obtained with different adherences rates of drug A and B, in absence of a true effect (RR_true_ = 1.0). Observed relative risk for different levels of adherence to escitalopram (80%) compared paroxetine (60%) was 1.08, assuming a relation of RR 1.5 of both antidepressant agents with the outcome

Differences in levels of adherence, however, did generate RRs deviating from 1.0, in the absence of a difference between Drug A and Drug B (Table 5). The different adherence rates required to observe an RR of 0.80, 0.90, 1.10, or 1.25 are shown in Figure 6. For example, 80% and 64% adherence for Drugs A and B, both with an RR of 2.0 with the outcome, resulted in an observed RR_A‐B_ of 1.10. Applying this to the comparison between escitalopram (80% adherence) and paroxetine (60% adherence), both with an RR of 1.5 with the outcome, an RR of 1.08 could have been observed when comparing escitalopram to paroxetine. Additional figures are presented in Figure S3 for scenarios where both drugs had a stronger relation with the outcome (RR 5.0 and 10.0).

**TABLE 5:** Impact of different values of nonadherence in comparative effectiveness research

	Truth		Observed data	
Paroxetine use versus nonusers	Drug P1	Drug P0	Drug P1	Drug P0
Y = 1	150	9900	217	9833
Y = 0	850	89 100	1450	88 500
Total	1000	99 000	1667	98 333
Risk	0.15	0.10	0.13	0.10
RR	1.5		1.4	
Escitalopram versus nonusers	Drug E1	Drug E0	Drug E1	Drug E0
Y = 1	75	9950	87.5	9937.5
Y = 0	425	89 550	537.5	89437.5
Total	500	99 500	625	99 375
Risk	0.15	0.10	0.14	0.10
RR	1.5		1.8	
Escitalopram versus paroxetine	Drug P1	Drug E1	Drug P1	Drug E1
Y = 1	150	75	217	87.5
Y = 0	850	425	145	537.5
Total	1000	500	1667	625
Risk	0.15	0.15	0.13	0.14
RR_E−P_	1.0		1.08	

*Note*: Values used: baseline risk: 0.1; RR: paroxetine 1.5, escitalopram 1.5; exposure prevalence: paroxetine 0.01, escitalopram 0.005; data capture: paroxetine 1.0, escitalopram 1.0; adherence: paroxetine 0.6, escitalopram 0.8.

Abbreviations: P1: exposed to paroxetine; E1 exposed to escitalopram; P0 and E0 nonexposed to paroxetine or escitalopram; Y = outcome.

## 6 DISCUSSION AND CONCLUSION

We studied the impact of a range of different values for nonadherence and uncaptured exposure to understand the relative impact of those two sources of exposure misclassification. Among the scenarios considered, we found that for exposure with a prevalence of less than 40%–50%, nonadherence had a greater impact on the RR than uncaptured exposure. To put this in context, in pharmacoepidemiology, the exposure prevalence for most drugs is <10%, unless studies are restricted to those with an indication for the drug, such as exposure to antidepressants within patients diagnosed with depression.

For an exposure with a prevalence of 10%, 25% nondifferential nonadherence changed the RR from 2.0 to 1.75, while applying 25% nondifferential uncaptured exposure changed the RR from 2.0 to 1.95. A substantial degree of nonadherence can therefore lead to associations being missed, especially if the effect under study is small. Applying nondifferential exposure misclassification to the examples of NSAIDs and SSRIs and the risk of bleeding, we demonstrated that an attenuation of ±20%–30% of the true relative risk can be expected using the values for nonadherence and uncaptured data of antidepressant drugs and NSAIDs, as shown in Table 2. However, these percentages of attenuation are not fixed values, but an example of the degree of bias that can be expected. A range of values has been described in the literature for the degree of nonadherence and uncaptured data, and we used one of many possible combinations. In addition, these scenarios may turn out differently for different databases, as there are varying reasons per database why exposure status can be misclassified (Table 1).

The impact of uncaptured exposure was dependent on exposure prevalence, since uncaptured exposure changed the observed risk of the unexposed group without changing the observed risk among the exposed. The larger the group of truly unexposed was, the smaller the effect of uncaptured exposure was. This was not seen for the effect on nonadherence: in this case, the observed risk of the exposed subjects was changed by misclassifying unexposed subjects as exposed, but nonadherence had no impact on the observed risk of the unexposed. Therefore, the effect of nonadherence was not impacted by exposure prevalence.

Studying the effect of differential misclassification, we found in this simulation RRs deviating away from the null. However, the differences in captured data or adherence between drug users with and without a risk factor with a relative risk of 2.0 with the outcome needed to be large (e.g., 50% vs. 90%) to result in a clinically relevant deviation from the null (arbitrarily set at RR_obs_ 1.10). This has also been demonstrated in the NSAID case and differential misclassification caused by age. In this specific example, the bias can be removed because age is often corrected for in the analysis. However, there are also examples of unmeasured risk factors, such as smoking status, which can lead to biased results if this risk factor is related to both the outcome and the risk of exposure misclassification. In addition, in the studied scenarios, the risk factor was present in 50% of all subjects. However, with a different distribution of the risk factor (e.g., 10% or 90%), the effect of the differential exposure misclassification was even smaller, and the differences between subjects with and without the risk factor needed to be larger to result in a clinically relevant deviation (Figures S1 and S2).

In a comparative study of Drug A versus Drug B, the proportion of uncaptured drug exposure (nondifferential) had no impact on the effect estimates, since including only the captured exposure involved the same process as random sampling, as long as the misclassification due to uncaptured data was nondifferential. Different levels of adherence between Drugs A and B could lead to the estimates of Drug A versus Drug B deviating from the null in the absence of a true different effect. In this case, however, the exposure definition is not dichotomous, but polytomous (exposed to A, B, or none), and it has already been shown that nondifferential misclassification of a polytomous exposure can cause bias away or toward the null.^52^ In addition, the differences in adherence needed to be large (e.g., 80% and 64%) to result in a clinically relevant deviation from the null effect (RR_obs_ 1.10), when both drugs had an RR of 2.0 on the outcome, or 80% and 55% when both drugs had an RR of 1.5 on the outcome.

These conclusions are in contrast to prior literature, which has demonstrated that small violations of the assumption of misclassification being nondifferential or differences in misclassification between Drugs A and B could already result in clinically relevant deviations from the null effect.^22^, ^24^, ^53^ In these previous studies, misclassification was introduced by choosing different values for specificity and sensitivity, while we focused on values for nonadherence and uncaptured data. For example, Brenner used a sensitivity of 0.9 and a specificity of 0.9 for the exposure measurement, with an exposure prevalence of 0.01, 0.1, and 0.5. However, the degree of nonadherence required to result in these values of specificity is 91.5%, 50%, and 10% for the different exposure prevalences, respectively. Since most drugs have an exposure prevalence of up to 10%, and nonadherence is often <40%, we considered a specificity of 0.9 to be unlikely for current pharmacoepidemiologic database studies. In the study by Jonsson‐Funk and Landi, chosen values for the misclassification of Drug A usage versus nonuse were a sensitivity of 0.85 and a specificity 0.95 (exposure prevalence 0.17), and for Drug B usage versus nonuse, the values were a sensitivity of 0.90 and a specificity of 0.98 (exposure prevalence 0.02). The RR observed for this scenario was 1.20 instead of a true effect of 1.0. In our study, these values translate to 23% and 53% nonadherence, respectively; hence, nonadherence to Drug B was 2.3 times higher compared to Drug A. The strength of our study was that we used literature‐based values for nonadherence and uncaptured data, which helps to contextualize the results and enables other researchers to apply these values in their own research.

Another strength of this study was that we were able to examine the effects of both uncaptured exposure and nonadherence in one model. This provided insight into which source of misclassification of exposure has the greatest impact. A limitation of this study was that the model we used was a simplification of the true mechanisms causing exposure misclassification. For example, in the simple 2 × 2 tables, time effects were ignored in the analysis, such as the fact that subjects prone to a negative outcome quit using the drug earlier than subjects who tolerated the drug better. We also ignored the fact that dosages and associated risks could differ between captured and uncaptured exposure, which is the case, for example, for prescription NSAID use versus OTC use and the risk of bleeding.^54^, ^55^

To conclude, in all scenarios studied, the values for nonadherence and uncaptured data or the differences in these values between subgroups needed to be relatively large to lead to clinically relevant bias. With estimates of the degree of misclassification, for example from pilot studies or published results of drug utilization research, a simple bias analysis can provide insight into the impact of exposure misclassification on the effect estimates. Therefore, we provide additional tables and figures in Appendix S1, which can be used to assess the impact of the different sources of misclassification, using values for exposure prevalence, the proportion of nonadherence, and uncaptured data.

It should be kept in mind that scenarios may turn out differently for different databases, as depicted in Figure 1. A prescription‐only drug that is fully reimbursed is likely to have nearly 100% captured exposure in a claims database, but a lower percentage in a single prescriber database, when there are multiple prescribers. Nonadherence can also have a different effect in prescribing and dispensing databases, depending on whether a subject decides not to collect the prescribed drug or decides not to ingest the drug after collecting it. We therefore recommend that authors provide estimates of the degree of exposure misclassification, instead of only a vague statement about the possibility of such misclassification, and to use the values of nonadherence and uncaptured exposure in sensitivity analyses to test the robustness of findings.

## CONFLICT OF INTEREST

No authors report any conflict of interest.

## ETHICS STATEMENT

The authors state that no ethical approval was needed.

## Supporting information


**Appendix S1:** Supporting Information
