# Supplementary material for: Drug exposure misclassification in pharmacoepidemiology: Sources and relative impact
Source: Pharmacoepidemiol Drug Saf. 2021 Sep 7;30(12):1703–15. doi: 10.1002/pds.5346 (PMC9292927; doi:10.1002/pds.5346)

Content

Table S1 - Non-differential exposure misclassification 2

Table S1a - Observed RR 1.10 2

Table S1b - Observed RR 1.25 3

Table S1c - Observed RR 1.50 4

Table S1d - Observed RR 2.0 5

Figure S1 - Differential exposure misclassification – uncaptured exposures 6

Figure S1a – Effect of differential uncaptured exposures - risk factor present in 10% of subjects 6

Figure S1b – Effect of differential uncaptured exposures - risk factor present in 25% of subjects 7

Figure S1c – Effect of differential uncaptured exposures - risk factor present in 50% of subjects 8

Figure S2 - Differential exposure misclassification – non-adherence 9

Figure S2a– Effect of differential non-adherence - risk factor present in 10% of subjects 9

Figure S2b– Effect of differential non-adherence - risk factor present in 25% of subjects 10

Figure S2c– Effect of differential non-adherence - risk factor present in 50% of subjects 11

Figure S3 - Comparative effectiveness research (drug A vs B) 12

Table S1 - Non-differential exposure misclassification

Table S1a - Observed RR 1.10

True relative risks that would lead to an observed relative risk of 1.10 in presence of different levels of uncaptured exposure or non-adherence

**Amount of misclassification**		**True RR for different exposure prevalences**		
Prop non-adherence	Prop uncap-tured data	p=0.01	p=0.10	p=0.25
0.0	0.0	1.10	1.10	1.10
0.0	0.1	1.10	1.10	1.10
0.0	0.2	1.10	1.10	1.11
0.0	0.3	1.10	1.10	1.11
0.0	0.4	1.10	1.10	1.11
0.0	0.5	1.10	1.11	1.12
0.1	0.0	1.11	1.11	1.11
0.1	0.1	1.11	1.11	1.12
0.1	0.2	1.11	1.11	1.12
0.1	0.3	1.11	1.12	1.13
0.1	0.4	1.11	1.12	1.13
0.1	0.5	1.11	1.12	1.13
0.2	0.0	1.12	1.12	1.12
0.2	0.1	1.12	1.13	1.13
0.2	0.2	1.13	1.13	1.14
0.2	0.3	1.13	1.13	1.14
0.2	0.4	1.13	1.13	1.15
0.2	0.5	1.13	1.13	1.16
0.3	0.0	1.14	1.14	1.14
0.3	0.1	1.14	1.15	1.15
0.3	0.2	1.14	1.15	1.16
0.3	0.3	1.14	1.15	1.17
0.3	0.4	1.14	1.15	1.18
0.3	0.5	1.14	1.16	1.19
0.4	0.0	1.17	1.17	1.17
0.4	0.1	1.17	1.17	1.18
0.4	0.2	1.17	1.17	1.19
0.4	0.3	1.17	1.18	1.21
0.4	0.4	1.17	1.18	1.22
0.4	0.5	1.17	1.18	1.23
0.5	0.0	1.20	1.20	1.20
0.5	0.1	1.20	1.20	1.22
0.5	0.2	1.20	1.21	1.24
0.5	0.3	1.20	1.22	1.27
0.5	0.4	1.20	1.22	1.29
0.5	0.5	1.20	1.23	1.31

Table S1b - Observed RR 1.25
True relative risks that would lead to an observed relative risk of 1.25 in presence of different levels of uncaptured exposure or non-adherence

**Amount of misclassification**		**True RR for different exposure prevalences**		
Prop non-adherence	Prop uncap-tured data	p=0.01	p=0.10	p=0.25
0.0	0.0	1.25	1.25	1.25
0.0	0.1	1.25	1.25	1.26
0.0	0.2	1.25	1.26	1.27
0.0	0.3	1.25	1.26	1.28
0.0	0.4	1.25	1.26	1.29
0.0	0.5	1.25	1.27	1.30
0.1	0.0	1.28	1.28	1.28
0.1	0.1	1.28	1.28	1.29
0.1	0.2	1.28	1.29	1.30
0.1	0.3	1.28	1.29	1.32
0.1	0.4	1.28	1.30	1.33
0.1	0.5	1.28	1.30	1.35
0.2	0.0	1.31	1.31	1.31
0.2	0.1	1.31	1.32	1.33
0.2	0.2	1.31	1.32	1.35
0.2	0.3	1.31	1.33	1.37
0.2	0.4	1.31	1.33	1.39
0.2	0.5	1.31	1.34	1.41
0.3	0.0	1.36	1.36	1.36
0.3	0.1	1.36	1.36	1.38
0.3	0.2	1.36	1.37	1.41
0.3	0.3	1.36	1.38	1.43
0.3	0.4	1.36	1.39	1.46
0.3	0.5	1.36	1.39	1.49
0.4	0.0	1.42	1.42	1.42
0.4	0.1	1.42	1.43	1.45
0.4	0.2	1.42	1.44	1.49
0.4	0.3	1.42	1.45	1.53
0.4	0.4	1.42	1.46	1.58
0.4	0.5	1.42	1.47	1.62
0.5	0.0	1.50	1.50	1.50
0.5	0.1	1.50	1.51	1.56
0.5	0.2	1.50	1.53	1.63
0.5	0.3	1.50	1.55	1.70
0.5	0.4	1.50	1.56	1.78
0.5	0.5	1.51	1.58	1.85

Table S1c - Observed RR 1.50

True relative risks that would lead to an observed relative risk of 1.50 in presence of different levels of uncaptured exposure or non-adherence

**Amount of misclassification**		**True RR for different exposure prevalences**		
Prop non-adherence	Prop uncap-tured data	p=0.01	p=0.10	p=0.25
0.0	0.0	1.50	1.50	1.50
0.0	0.1	1.50	1.51	1.53
0.0	0.2	1.50	1.52	1.55
0.0	0.3	1.50	1.53	1.58
0.0	0.4	1.50	1.53	1.61
0.0	0.5	1.50	1.54	1.64
0.1	0.0	1.56	1.56	1.56
0.1	0.1	1.56	1.57	1.59
0.1	0.2	1.56	1.58	1.62
0.1	0.3	1.56	1.59	1.66
0.1	0.4	1.56	1.60	1.69
0.1	0.5	1.56	1.61	1.73
0.2	0.0	1.62	1.62	1.62
0.2	0.1	1.63	1.64	1.67
0.2	0.2	1.63	1.65	1.71
0.2	0.3	1.63	1.67	1.76
0.2	0.4	1.63	1.68	1.81
0.2	0.5	1.63	1.69	1.86
0.3	0.0	1.71	1.71	1.71
0.3	0.1	1.72	1.73	1.77
0.3	0.2	1.72	1.75	1.84
0.3	0.3	1.72	1.77	1.91
0.3	0.4	1.72	1.79	1.98
0.3	0.5	1.72	1.81	2.06
0.4	0.0	1.83	1.83	1.83
0.4	0.1	1.83	1.86	1.93
0.4	0.2	1.84	1.88	2.02
0.4	0.3	1.84	1.91	2.13
0.4	0.4	1.84	1.94	2.25
0.4	0.5	1.84	1.96	2.37
0.5	0.0	2.00	2.00	2.00
0.5	0.1	2.00	2.04	2.16
0.5	0.2	2.01	2.08	2.33
0.5	0.3	2.01	2.12	2.53
0.5	0.4	2.01	2.16	2.75
0.5	0.5	2.01	2.20	3.00

Table S1d - Observed RR 2.0
True relative risks that would lead to an observed relative risk of 2.0 in presence of different levels of uncaptured exposure or non-adherence

**Amount of misclassification**		**True RR for different exposure prevalences**		
Prop non-adherence	Prop uncap-tured data	p=0.01	p=0.10	p=0.25
0.0	0.0	2.00	2.00	2.00
0.0	0.1	2.00	2.02	2.07
0.0	0.2	2.00	2.05	2.14
0.0	0.3	2.01	2.07	2.22
0.0	0.4	2.01	2.09	2.31
0.0	0.5	2.01	2.12	2.40
0.1	0.0	2.11	2.11	2.11
0.1	0.1	2.11	2.14	2.20
0.1	0.2	2.12	2.17	2.30
0.1	0.3	2.12	2.20	2.40
0.1	0.4	2.12	2.23	2.51
0.1	0.5	2.12	2.26	2.64
0.2	0.0	2.25	2.25	2.25
0.2	0.1	2.25	2.29	2.37
0.2	0.2	2.26	2.32	2.50
0.2	0.3	2.26	2.36	2.64
0.2	0.4	2.26	2.40	2.80
0.2	0.5	2.27	2.44	2.98
0.3	0.0	2.43	2.43	2.43
0.3	0.1	2.43	2.48	2.60
0.3	0.2	2.44	2.53	2.78
0.3	0.3	2.44	2.58	3.00
0.3	0.4	2.44	2.63	3.24
0.3	0.5	2.45	2.69	3.52
0.4	0.0	2.67	2.67	2.67
0.4	0.1	2.67	2.73	2.92
0.4	0.2	2.68	2.80	3.22
0.4	0.3	2.68	2.88	3.57
0.4	0.4	2.69	2.96	4.00
0.4	0.5	2.69	3.04	4.51
0.5	0.0	3.00	3.00	3.00
0.5	0.1	3.01	3.10	3.44
0.5	0.2	3.02	3.21	4.00
0.5	0.3	3.02	3.32	4.71
0.5	0.4	3.03	3.44	5.66
0.5	0.5	3.04	3.57	6.99

Figure S1 - Differential exposure misclassification – uncaptured exposures

Figure S1a – Effect of differential uncaptured exposures - risk factor present in 10% of subjects

Observed relative risks obtained with different proportion of exposure captured for subjects with and without risk factor, in absence of a true effect (RR_{true} = 1.0).

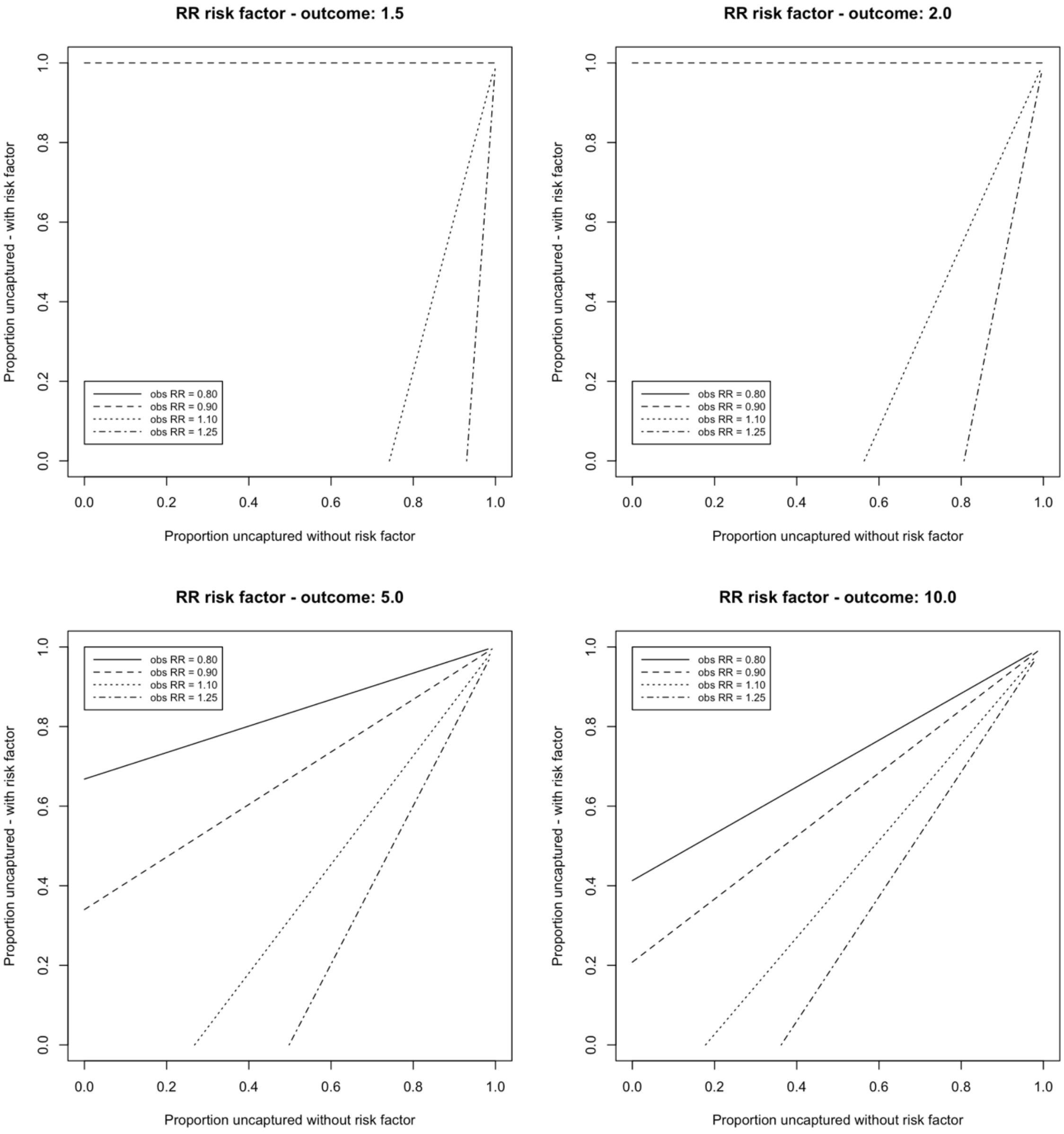

Figure S1b – Effect of differential uncaptured exposures - risk factor present in 25% of subjects
Observed relative risks obtained with different proportion of exposure captured for subjects with and without risk factor, in absence of a true effect (RR_{true} = 1.0).

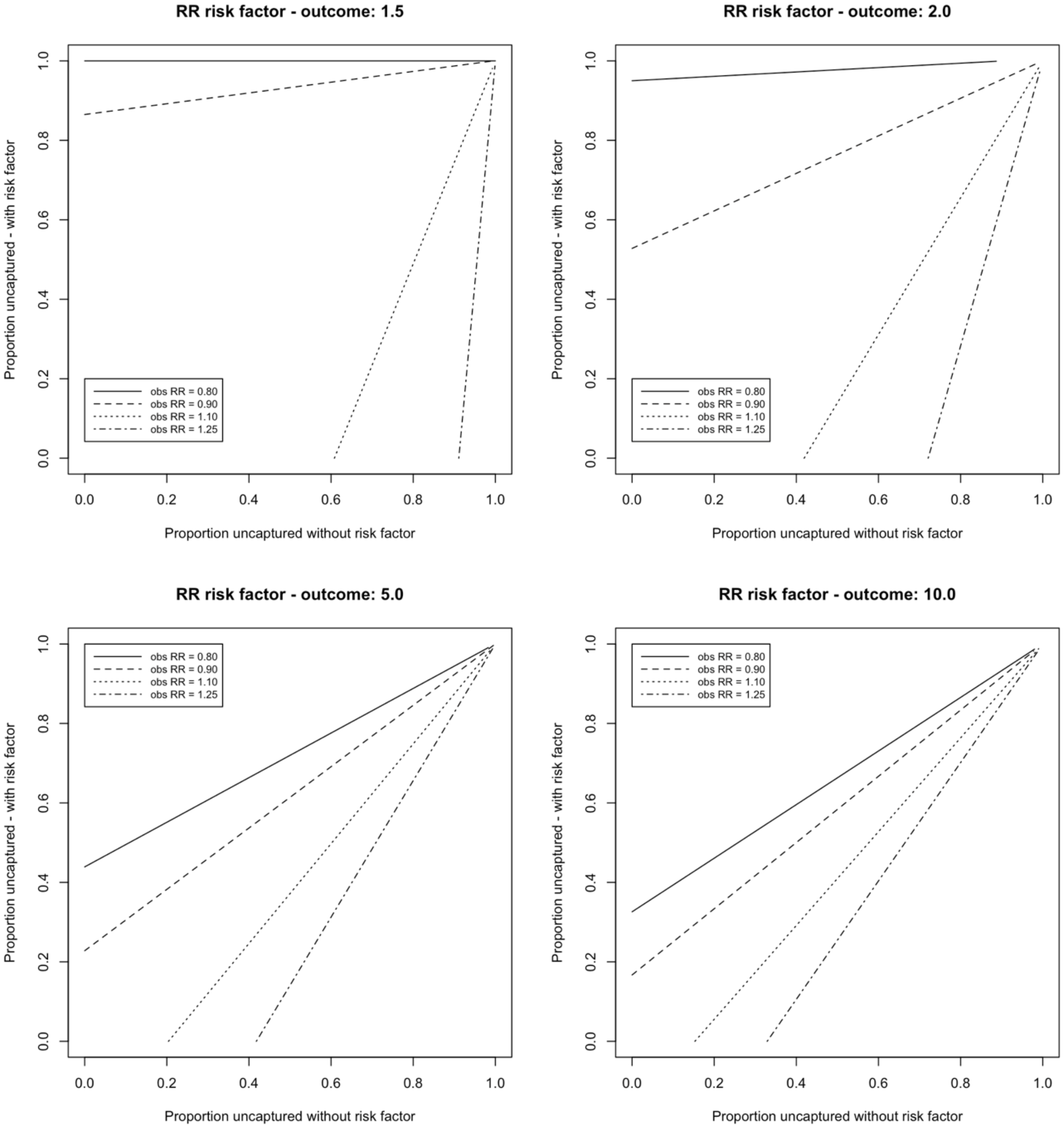

Figure S1c – Effect of differential uncaptured exposures - risk factor present in 50% of subjects
Observed relative risks obtained with different proportion of exposure captured for subjects with and without risk factor, in absence of a true effect (RR_{true} = 1.0).

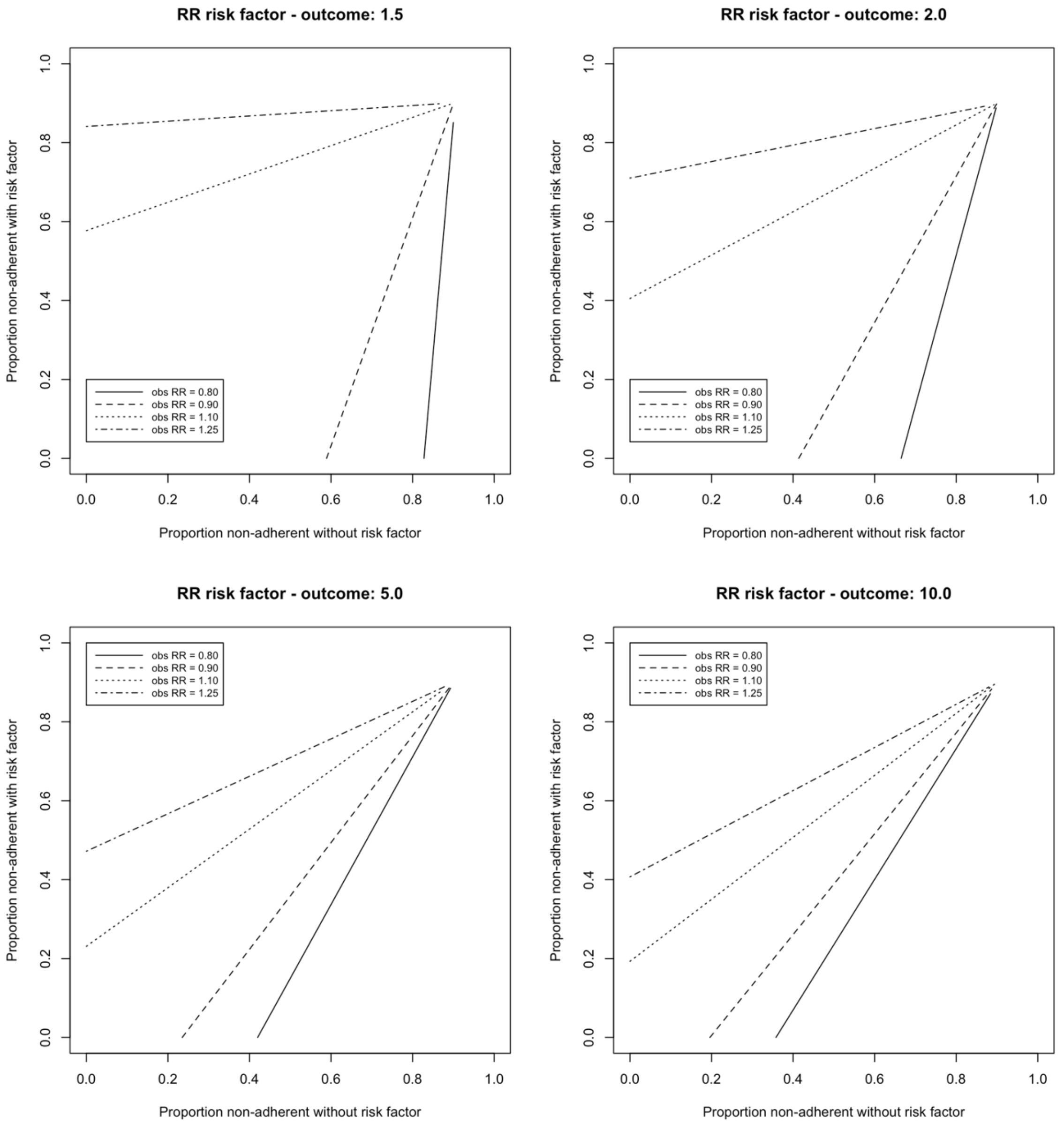

Figure S2 - Differential exposure misclassification – non-adherence

Figure S2a– Effect of differential non-adherence - risk factor present in 10% of subjects
Observed relative risks obtained with different proportion of exposure captured for subjects with and without risk factor, in absence of a true effect (RR_{true} = 1.0).

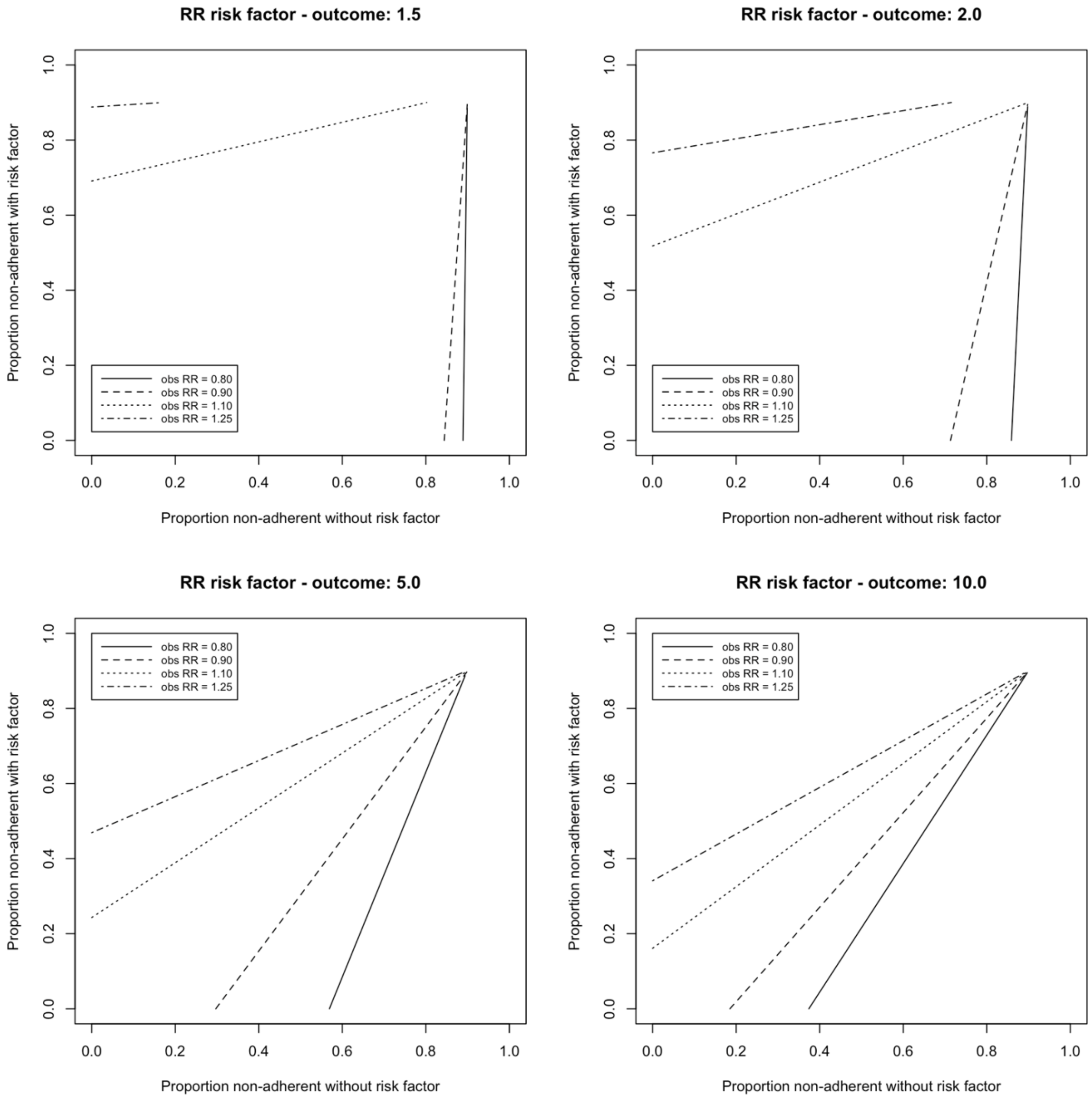

Figure S2b– Effect of differential non-adherence - risk factor present in 25% of subjects
Observed relative risks obtained with different proportion of exposure captured for subjects with and without risk factor, in absence of a true effect (RR_{true} = 1.0).

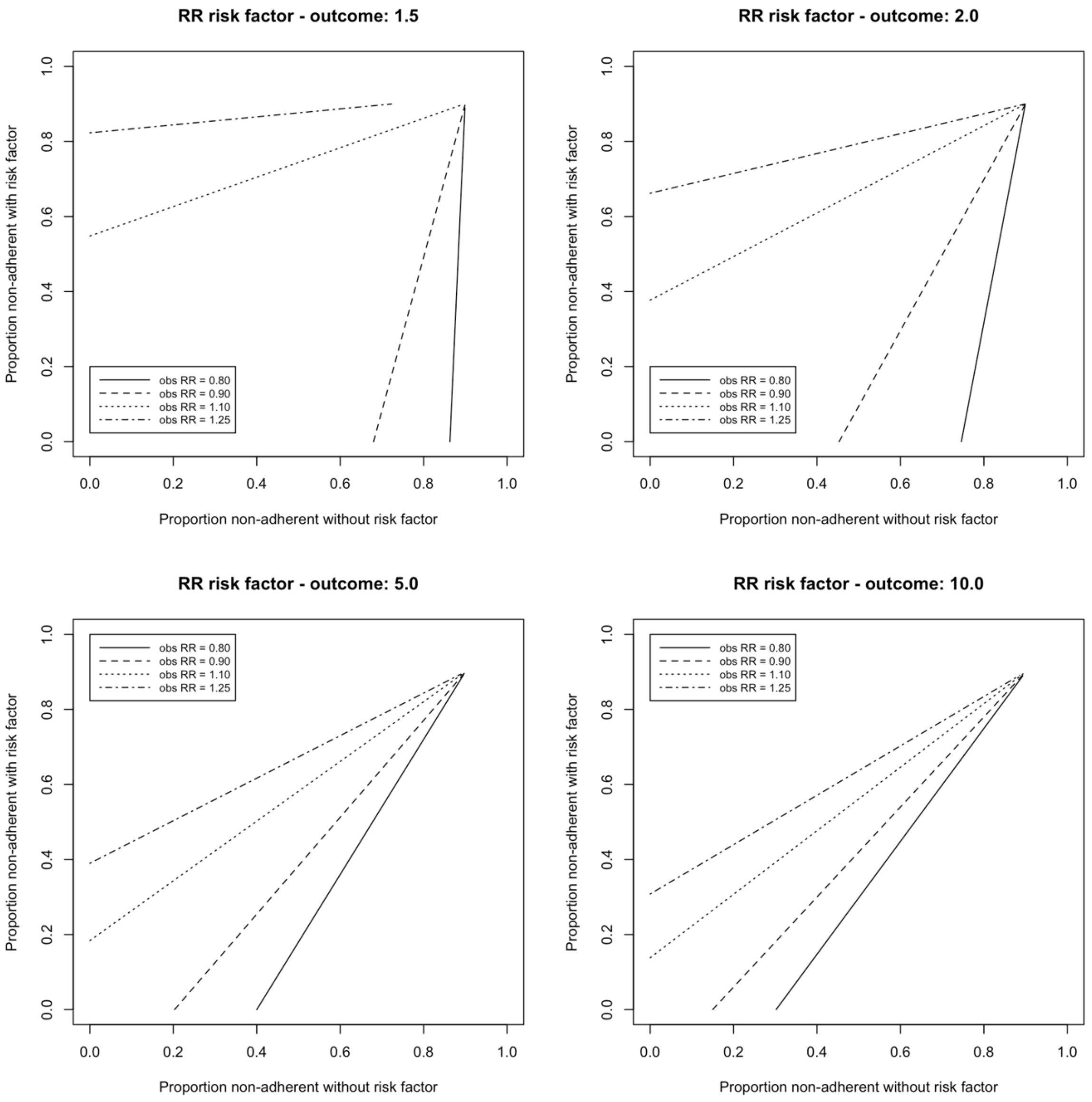

Figure S2c– Effect of differential non-adherence - risk factor present in 50% of subjects
Observed relative risks obtained with different proportion of exposure captured for subjects with and without risk factor, in absence of a true effect (RR_{true} = 1.0).

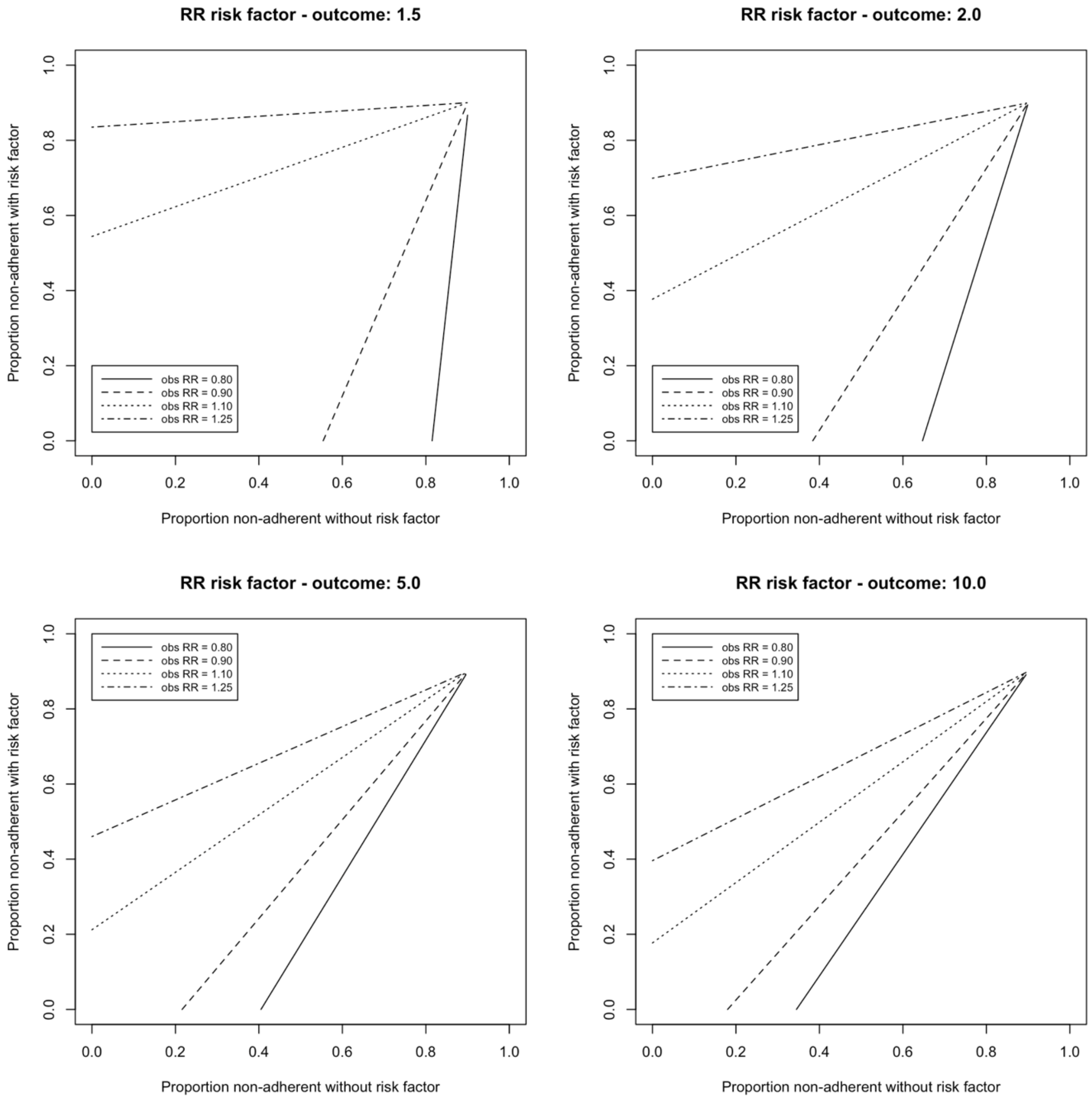

Figure S3 - Comparative effectiveness research (drug A vs B)

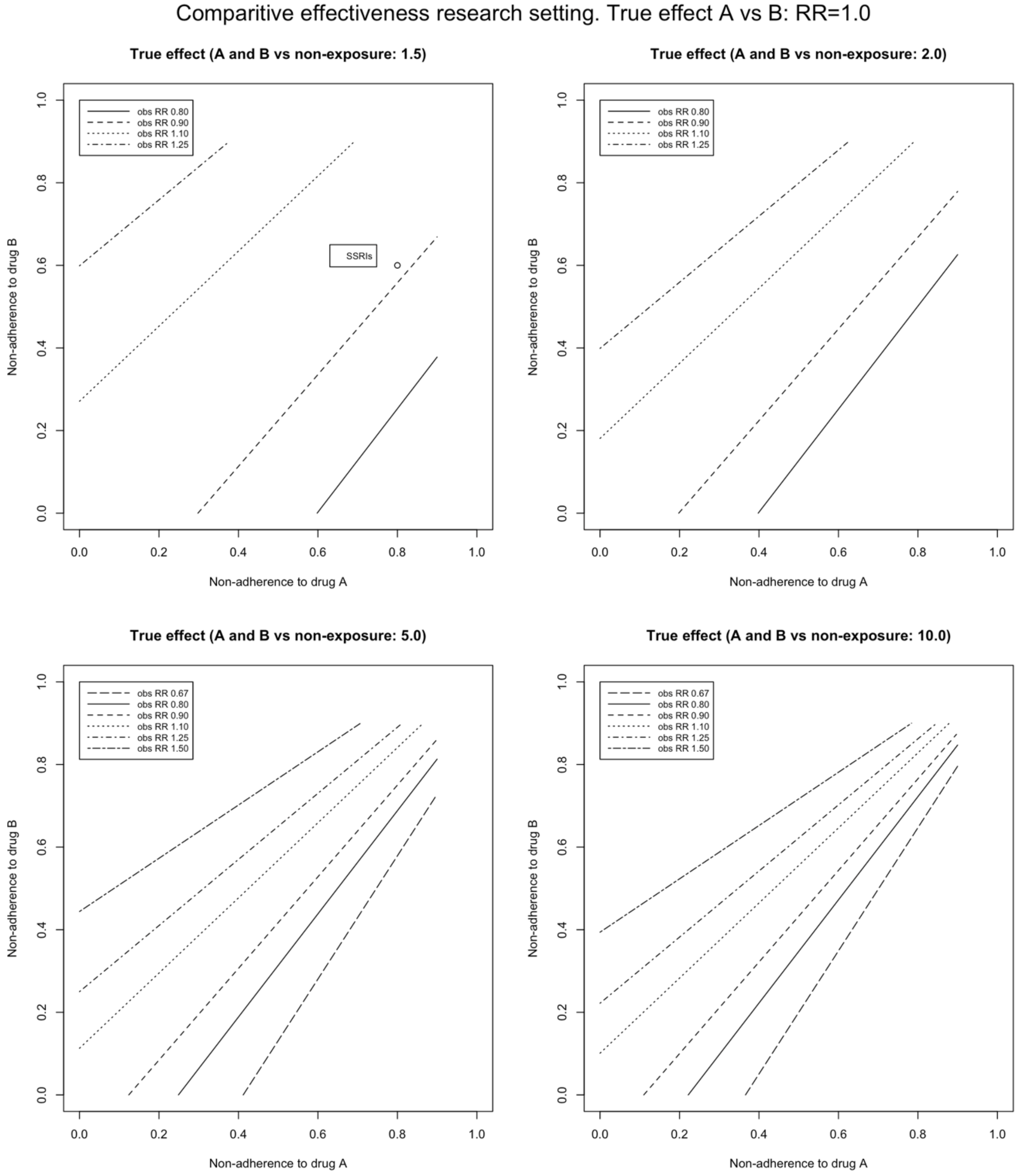

Supplement: Supplementary file 1 — Appendix S1: Supporting Information [file PDS-30-1703-s001.pdf]
